# HIV-1 Pre-Integration Complexes Selectively Target Decondensed Chromatin in the Nuclear Periphery

**DOI:** 10.1371/journal.pone.0002413

**Published:** 2008-06-11

**Authors:** Alberto Albanese, Daniele Arosio, Mariaelena Terreni, Anna Cereseto

**Affiliations:** 1 Laboratory of Molecular Biology Scuola Normale Superiore, Pisa, Italy; 2 NEST, CNR-INFM and Scuola Normale Superiore, Pisa, Italy; University of Arkansas, United States of America

## Abstract

Integration of the double-stranded DNA copy of the HIV-1 genome into host chromosomal DNA is a requirement for efficient viral replication. Integration preferentially occurs within active transcription units, however chromosomal site specificity does not correlate with any strong primary sequence. To investigate whether the nuclear architecture may affect viral integration we have developed an experimental system where HIV-1 viral particles can be visualized within the nuclear compartment. Fluorescently labeled HIV-1 virions were engineered by fusing integrase, the viral protein that catalyzes the integration reaction, to fluorescent proteins. Viral tests demonstrate that the infectivity of fluorescent virions, including the integration step, is not altered as compared to wild-type virus. 3-D confocal microscopy allowed a detailed analysis of the spatial and temporal distribution of the pre-integration complexes (PICs) within the nucleus at different moments following infection; the fluorescently labeled PICs preferentially distribute in decondensed areas of the chromatin with a striking positioning in the nuclear periphery, while heterochromatin regions are largely disfavored. These observations provide a first indication of how the nuclear architecture may initially orient the selection of retroviral integration sites.

## Introduction

HIV-1 to efficiently complete a replication cycle has to integrate its genome into the host cellular DNA. This reaction is catalyzed by the virus-encoded protein integrase (IN). IN together with other viral and cellular proteins forms the pre-integration complex (PIC) and binds specific sequences located at the ends of the viral cDNA (*att* sites) [1]. So far no primary sequence in the cellular genome has been identified as the preferential binding site for IN during viral integration, though a weakly conserved palindromic sequence has been identified [2]–[5]. Notwithstanding the lack of strong sequence specificity, recent evidence suggests that retroviral integration does not occur at random in DNA molecules. Indeed, it has been demonstrated that the majority of retroviruses preferentially integrates in DNase I hypersensitive regions [6]–[10] and in transcriptionally-active chromosomal regions [11]–[16]. Symmetrically, other authors showed that centromeric alphoid-repeat regions are disfavored integration sites [2]. Noteworthy, DNase hypersensitive regions and transcriptionally active genes are found in open-decondensed chromatin regions, in contrast with alpha repeats which are known to localize in closed-condensed chromatin regions. These observations together with *in vitro* data [17] suggest that chromatin structure may represent a determinant factor for target-site selection during retroviral integration. In eukaryotic cells DNA is assembled with nucleosomes to form chromatin, which assumes at least two distinct structural and functional forms: a condensed form that generally lacks DNA regulatory activity and a looser, decondensed form that provides the environment for DNA regulatory processes such as DNA replication, repairs and transcription. Chromatin is compartmentalized within the nucleus under a specific nuclear architecture.

HIV-1 replication has been extensively studied using a wide array of molecular biology, biochemistry and structural biology approaches. However, in order to verify a role of the chromatin structure and distribution during the viral life cycle is critical to directly visualize the virus inside intact nuclei of infected cells. Therefore, we have developed an experimental system that, by exploiting the power of three-dimensional optical dissection of sub-cellular structures, would be potentially able to simultaneously visualize HIV-1 particles and chromatin organization within intact nuclei.

To this aim, we have engineered viral particles containing IN fused to the Enhanced Cyan/Green Fluorescent Protein (IN-ECFP/IN-EGFP) through the “trans-incorporation” technique. This method exploits the Vpr property to shuttle fused exogenous proteins inside the viral particles [18]. This approach was successfully used to produce HIV-1 infectious particles containing a functional IN fused to LexA [19]. We demonstrate that the IN-EGFP containing virions are infectious and can be visualized within the nuclear compartment. To establish the intranuclear localization of the fluorescent viral particles, we have exploited at least two methods for nuclear visualization: nuclear lamina immunostaining and expression of histones H2B fused to the Enhanced Yellow Fluorescent Protein (H2B-EYFP). This system has proven valid to analyze virus-nuclear structure interaction and brought us to the observation that PICs are non-randomly distributed at the nuclear level with respect to chromatin structure and nuclear architecture.

## Results

### Construction of HIV-1 containing IN-EGFP fusion proteins

The introduction of exogenous coding sequences into the provirus impairs the genome processing and consequently the synthesis of viral particles. Therefore, to incorporate IN fused to the chosen fluorophore (ECFP or EGFP) in HIV-1 virions we exploited the Vpr-*trans* method [18]. Vpr is incorporated into viral particles through its interaction with p6 of Gag; thus by fusing a non-viral protein to the C-terminus of Vpr the exogenous factor is shuttled inside the virions. As previously described [18] a HIV-1 sensitive proteolytic site has been introduced between Vpr and IN allowing the separation of the IN-EGFP fusion protein to catalyze the integration reaction. Pseudotyped HIV-1 viral stocks containing IN-EGFP (HIV-IN-EGFP) were prepared by expressing in 293T cells the Vpr-IN-EGFP fusion protein, the VSV-G envelope protein and the proviral construct pD64E. This HIV-1 clone is derived from an *env*-deleted pNL4.3 provirus expressing the luciferase reporter gene and a mutated IN protein. Since the substitution from Asp 64 to Glu suppresses IN catalytic activity, the IN-EGFP fusion protein provided in *trans* exclusively mediates the integration of the recombinant HIV-1 fluorescent virions.

**Figure 1:**
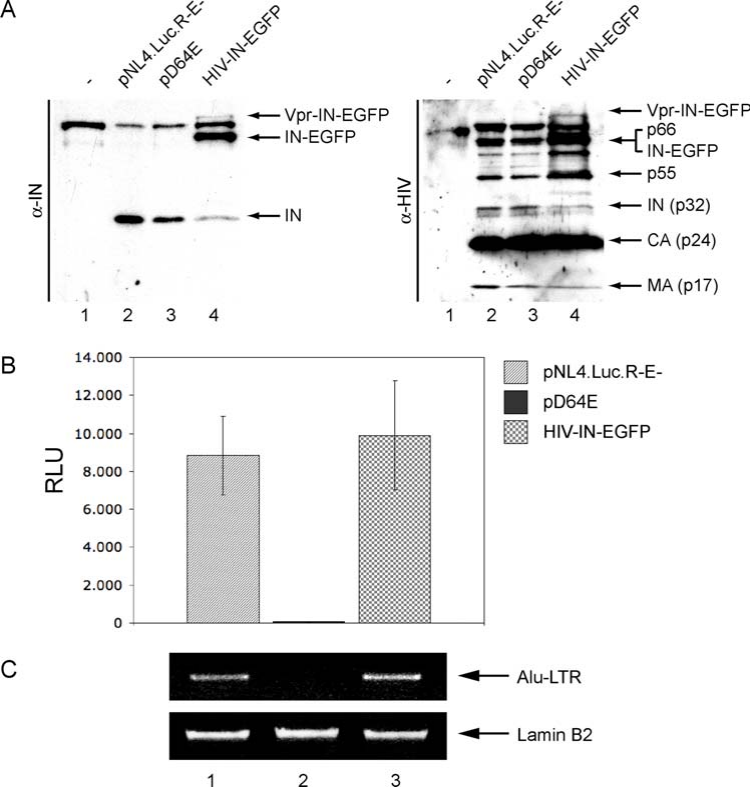
IN-EGFP trans-incorporation through Vpr produces intact and infective viral particles. (A) IN-EGFP is incorporated into intact HIV-1 virions. Supernatant from un-transfected control cells (lane 1) or cells transfected with pVSV-G and pNL4-3.Luc.R-E- (lane 2), pD64E (lane 3) or pD64E together with pVpr-IN-EGFP (lane 4) were pelleted by ultra-centrifugation through a 20% sucrose cushion and analyzed by western blot using antibodies anti-IN (left panel) or with an anti-HIV-1 human serum (right panel). (B) HIV-IN-EGFP virions are infective. Ultracentrifuged supernatants from cells producing the indicated viruses were normalized for p24 content and used to infect HeLa cells. Seven days post-infection cell lysates were analyzed for luciferase activity. Luciferase activity values were normalized for protein content of lysates and expressed as relative light units (RLU). (C) HIV-IN-EGFP virions integrate in the cellular genome. DNA extracted from HeLa cells infected for 24 hours with a NL4-3.Luc.R-E- (lane 1), D64E (lane 2) or HIV-IN-EGFP (lane 3) viruses pseudotyped with VSV-G were amplified with primers specific for the Alu-LTR (to analyze the fraction of integrated HIV-1 DNA-upper panel) and for the lamin B2 cellular gene (to standardize the total amount of extracted DNA- lower panel).

In order to determine whether the IN-EGFP fusion protein is efficiently incorporated into viral particles, concentrated viruses were analyzed by western blot. Using specific anti-IN antibodies, a band of the same size of IN (32 kDa) is detected in HIV-IN-EGFP virus as well as in control NL4-3.Luc.R-E- (wild-type), and D64E (mutated) viruses (Fig. 1A, left panel). In addition, two bands corresponding respectively to Vpr-IN-EGFP and to its proteolytic product IN-EGFP are exclusively visible in the fluorescently labeled virions (lane 4), proving that IN-EGFP is efficiently incorporated and is subsequently digested by the viral protease. Moreover, protein synthesis and maturation is not affected by Vpr-IN-EGFP *trans* incorporation, as indicated by an unmodified pattern of viral proteins visualized by an HIV-1 antiserum (Fig. 1A, right panel). The infectivity of IN-EGFP containing virions was then tested by measuring luciferase activity 7 days after infection. As expected, infection with the D64E virus produces very low levels of luciferase as compared to wild-type NL4-3.Luc.R-E- (Fig. 1B). Conversely, infection with the HIV-IN-EGFP virus showed luciferase activity at a level comparable to the wild type. This clearly demonstrates that IN-EGFP can efficiently *trans* complement the catalytically inactive IN expressed by the D64E provirus. Finally, in order to prove that the infectivity of recombinant fluorescent virions occurs through reconstituted integration capacity, quantification of integrated proviral DNA was assessed by Alu-PCR. As expected, integrated viral DNA was observed in cells infected with the NL4-3.Luc.R-E- and not with the D64E virus (Fig. 1C, upper panel, lanes 1 and 2); cells infected with the HIV-IN-EGFP virus (lane 3) showed levels of proviral DNA similar to wild-type, demonstrating that the *trans* incorporation of IN-EGFP protein has successfully recovers the integration process.

### Visualization of HIV-1 virions containing IN-EGFP

**Figure 2:**
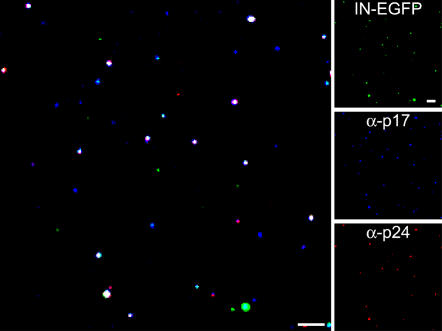
Visualization of HIV-IN-EGFP virions. Supernatants from cells transfected with the pD64E, pVpr-IN-EGFP and pVSV-G plasmids were ultra-centrifuged through a 20% sucrose cushion and adhered to glass coverslips. Following rinsing and fixation, immunostaining was performed with specific antibodies against viral proteins p17^MA^ and p24^CA^ and then visualized for EGFP fluorescence (green), p17^MA^ (blue) and p24^CA^ (red). Left panel is the merged image where white spots result from overlapping green, blue and red signals. Right panels show the individual fluorescent images. Bars, 5 µm.

**Figure 3:**
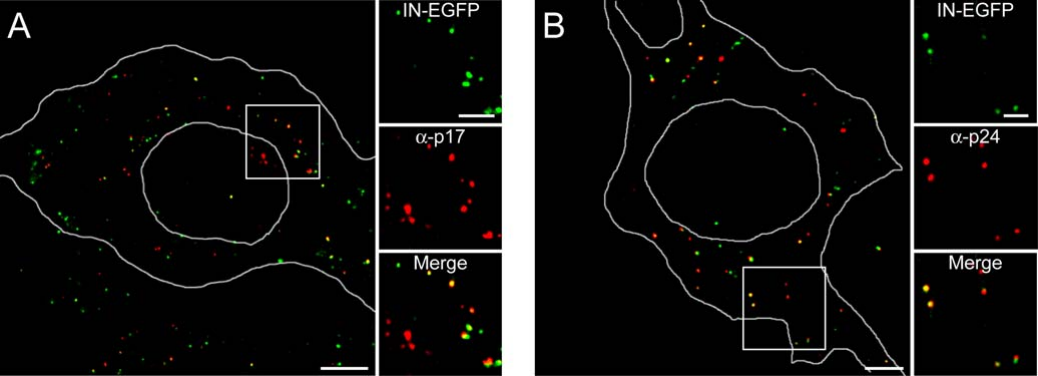
HIV-IN-EGFP virions can be visualized in infected cells. (A) Confocal visualization of HeLa cells infected with concentrated supernatants derived from cells transfected with the pD64E, pVpr-IN-EGFP and pVSV-G plasmids. Six hours post-infection cells were immunostained with antibodies against p17^MA^ and visualized for EGFP fluorescence (green) or p17^MA^ staining (red). In the merged image, where cell and nuclear shapes are outlined in white, yellow spots indicate full overlapping of green and red signals. Bar, 5 µm. Enlargements of the boxed region show individual color and merged images. Bar, 2 µm. (B) Same as in (A) using antibodies against p24^CA^.

The supernatant from 293T cells producing the HIV-IN-EGFP virus was concentrated by ultra-centrifugation and bound to glass coverslips. IN-EGFP containing viral particles were readily visualized and, consistently with former reports, they appeared heterogeneous in size and with a broad distribution of fluorescence intensity [20] (Fig. 2, upper right panel). To prove that the visualized fluorescent dots are indeed viral particles, the same viral supernatant was immunostained with antibodies recognizing the viral proteins matrix (p17^MA^) and capsid (p24^CA^) (Fig. 2, central- and bottom- right panels respectively). The merged image in Fig. 2, left panel, shows overlapping fluorescence signals (white dots) indicating that the majority of IN-EGFP virions are also positive for viral proteins. This analysis was also carried out in HeLa cells infected with viral supernatants. Point sources of EGFP fluorescence could be detected in the cells presumably marking the presence of viral PICs containing IN-EGFP. In order to verify the actual viral nature of the EGFP signals, cells were stained with antibodies against p17^MA^ and p24^CA^. Figure 3A shows that the majority of EGFP spots were positive for p17^MA^ and, consistently with Mc Donald et al. [21], colocalization was also observed with p24^CA^ (Fig. 3B).

**Figure 4:**
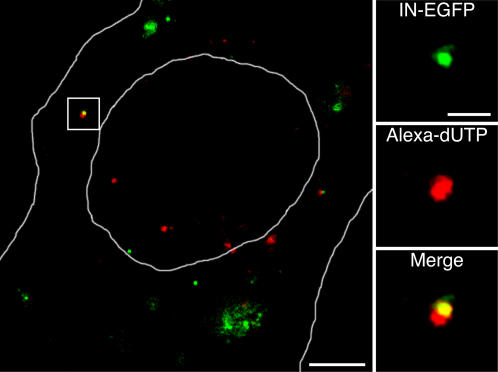
HIV-IN-EGFP virions containing neo-synthesized viral c-DNA can be visualized in infected cells. Confocal visualization of a HeLa cell at 6 hours after infection with HIV-IN-EGFP (green) viral supernatant incubated with deoxynucleotides Alexa-594-dUTP (red). The large panel shows the merged image where yellow indicates overlapping of green and red signals. Cell and nuclear shapes are outlined in white. Bar, 5 µm. Individual color and merged magnification images of the boxed region are shown in the right panels. Bar, 0.5 µm.

Functionally active PICs should associate with newly synthesized cDNA produced by reverse transcriptase (RT) activity. In order to verify whether the observed HIV-IN-EGFP PICs contains an intact RT, the natural endogenous reverse transcription (NERT) activity associated to extracellular virions was assessed by labeling and then visualizing the neo-synthesized cDNA. It was demonstrated that the NERT activity determines partial polymerization of the negative strand cDNA of infectious virions [22] and that this polymerization can be further activated by the addition of dNTPs [23], [24]. We incubated the HIV-IN-EGFP viral supernatant in the presence of fluorescently labeled deoxynucleotides (Alexa-594-dUTP) using appropriate conditions for viral RT activity. Following infection, EGFP labeled PICs were indeed found associated with fluorescently labeled cDNA (Fig. 4) demonstrating that IN-EGFP PICs contains a functionally active RT. Figure 4 shows, however, that only a minority of IN-EGFP spots overlap with fluorescently labeled cDNA. This reduced number of positive PICs can be explained by the rather low efficiency of the NERT assay and to the reported observation that only a limited number of entered viral particles is actually infective [25].

### Nuclear visualization of HIV-IN-EGFP PICs

IN is part of the viral PIC from the early steps of infection up to the integration event in the cellular genome. Thus, PICs containing IN fused to a fluorescent protein should be visible both in the cytoplasm and in the nuclear compartment. In order to specifically examine HIV-IN-EGFP PICs within the nuclear compartment, the nucleus of infected cells was visualized by immunostaining with antibodies recognizing the nuclear lamina (lamin A/C), which clearly defines the nucleus-cytoplasm boundary. Indeed, due to the irregularity of the nuclear membrane - determined by protrusions extending both inside and outside the nuclear surface – exact information on the 3D nuclear envelope shape are necessary to prove the nuclear localization of the observed PICs. In particular, it is necessary to define a spatial separation between the fluorescence sources of the signal deriving from the nuclear membrane and the one originating from the viral PICs. To this aim distorsions introduced by the microscope are removed by deconvolving z stacks containing IN-ECFP or IN-EGFP and lamin A/C Alexa-680 signals using experimentally determined point spread function (PSF) (see Methods).

**Figure 5:**
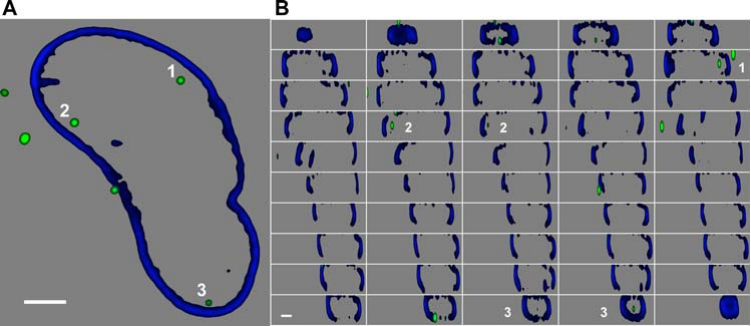
Nuclear visualization of the HIV-IN-ECFP virus. Nucleus of a HeLa cell infected with the HIV-IN-ECFP virus (green) and immunostained with lamin A/C (blue). Acquisition of confocal images was performed as z stack with a z step size of 0.3 µm and was followed by deconvolution based on experimentally determined PSF (see Experimental Procedure). (A) x-y section taken approximately at half high of the analyzed nucleus. (B) Adjacent vertical projections taken every 0.301 µm from upper (top-left panel) to lower side (bottom-right panel) of the nucleus in (A). Bars, 5 µm.

The real nuclear envelope profile (blue) and PICs (green) positions are thus assessed as illustrated in Fig. 5; panel A illustrates a single x-y section showing three distinct intranuclear PICs while panel B reports adjacent vertical nuclear sections derived from the same dataset. The three identified PICs (white labeled “1”, “2” and “3”) clearly localize in the inner part of the nucleus under study and are usually visible in two adjacent frames in line with the expected x-y resolution. This type of analysis allows excluding PICs localized in the outer proximity or juxtaposed to the nuclear membrane that could be mistakenly detected as intranuclear (see Supporting Information, Fig. S1).

**Figure 6:**
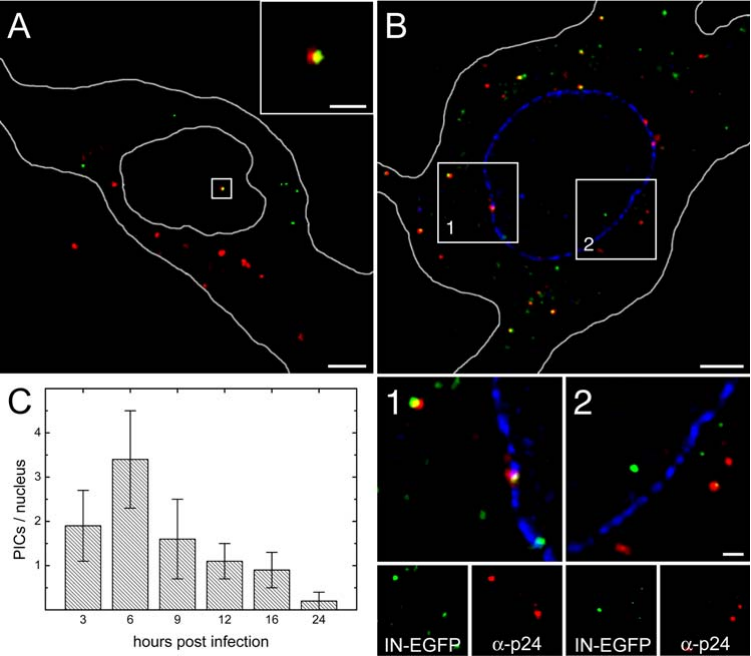
Nuclear HIV-IN-EGFP virions are functionally intact. (A) Merged image of a confocal section obtained from HeLa cells infected with HIV-IN-EGFP supernatants incubated with deoxynucleotides Alexa-594-dUTP. Yellow indicates full overlapping of EGFP and Alexa-594-dUTP signals. Cell and nuclear shapes are outlined in white. Bars, 5 µm and 1 µm in the whole frame and inset box, respectively. (B) Merged image of a confocal section obtained from HeLa cells infected with HIV-IN-EGFP supernatants (green) and immunostained with antibodies anti-p24^CA^ (red) and lamin A/C (blue). Yellow indicates full overlapping of EGFP and anti-p24^CA^ signals. Cell and nuclear shapes are outlined in white. Enlargements of frames 1 and 2 are shown in bottom panels along with the individual color images. Bars, 5 µM and 1 µm in the whole frame and enlarged boxes, respectively. (C) HeLa cells infected with HIV-IN-EGFP virions were fixed and the mean number of intranuclear viral particles per nucleus was quantified at 3 hrs, 6 hrs, 9 hrs, 12 hrs, 16 hrs and 24 hrs. At each time point an average of 30 cells was analyzed and SD represented as error bars.

To verify that the integrity of virions is preserved at the nuclear level, HeLa cells were infected with HIV-IN-EGFP viral stocks labeled with Alexa-594-dUTP by NERT as above described. Figure 6A shows the colocalization in the nucleus of IN-EGFP labeled PICs with neo-synthesized cDNA, proving that the fluorescent recombinant virus is indeed structurally intact at the nuclear level. Also, since virions translocate into the nucleus following p24^CA^ disassembly, the colocalization of p24^CA^ with nuclear HIV-IN-EGFP virions was analyzed. As shown in Fig. 6B the vast majority of PICs in the cytoplasm were positive for p24^CA^, while no PICs were stained for p24^CA^ in the nucleus, further proving the functional integrity of the nuclear HIV-IN-EGFP virions.

It was estimated that proviral DNA is detectable almost 8 hours after HIV-1 entry [26]. We have thus quantified the number of viral PICs at different time points from 3 to 24 hours after infection. Figure 6C shows that the maximum number of intranuclear HIV-IN-EGFP PICs (3.4±1.1; mean±SD; 30 cells) was observed after 6 hours. At subsequent times the number of PICs declined and virtually none was detected 24 hours after infection. Finally, compared to the total average amount of PICs per cell (167.5±33.4; mean±SD; 30 cells), at 6 hours post infection, only a small fraction (2.0±0.8%) was found in the nucleus.

### Distribution of HIV-1 particles in the nuclei of infected cells

**Figure 7:**
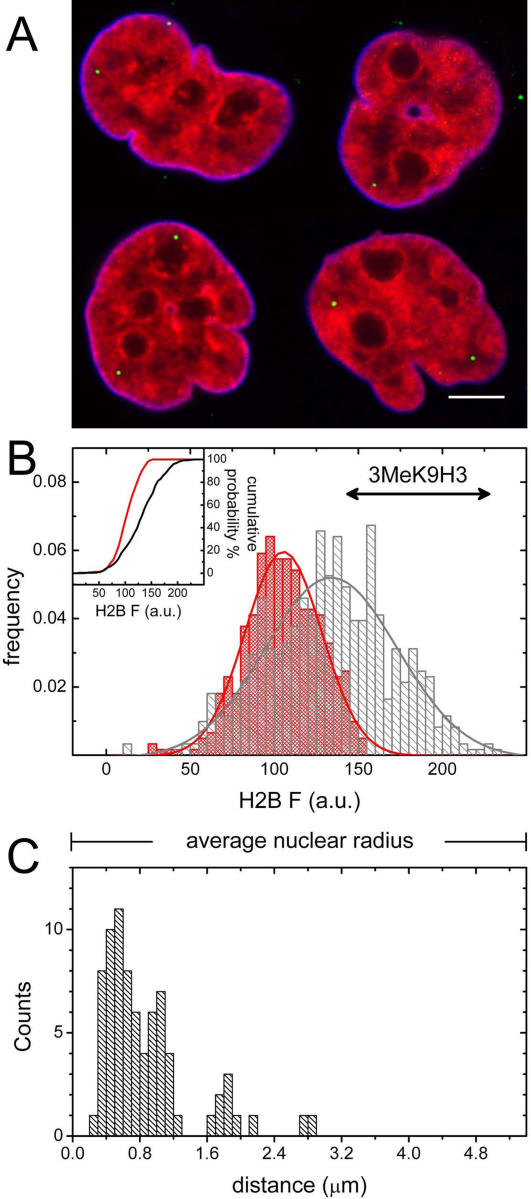
HIV-IN-ECFP virions preferentially localize in the periphery of the nucleus and outside heterochromatin regions. (A) Confocal images of HIV-IN-ECFP virions (green) in nuclei of HeLa cells expressing H2B-EYFP (red) and immunostained with antibody against lamin A/C (blue). Bar, 5 µm. (B) H2B-EYFP fluorescence intensity distribution frequency for PIC ROIs (defined by the HIV-IN-ECFP virions, red bars) and for Random ROIs (selected randomly in the same planes, grey bars) (n = 103). Solid lines are obtained by Gaussian fitting. The 3MeK9H3 labeled arrow represents the width at half-height of the H2B-EYFP fluorescence intensity distribution derived by cross analyzing with this heterochromatin marker (see Experimental Procedures). In the inset the distribution cumulative probabilities are plotted for PIC ROIs (red) and Random ROIs (grey) (*P*<0.001, Kolmogorov–Smirnov test). (C) Distribution of intranuclear PICs distances from the lamin A/C staining.

Chromatin assumes mainly two distinct conformations: condensed (heterochromatin) and decondensed (euchromatin). These configurations can be visualized in HeLa cells stably expressing histones H2B fused to EYFP (HeLa-H2B-EYFP) [27], where higher and lower EYFP fluorescence intensity areas indicate more and less condensed chromatin regions, respectively [28]–[30]. We exploited this experimental system to study HIV-1 compartmentalization with respect to chromatin structure. To this end HeLa-H2B-EYFP cells were infected with HIV-IN-ECFP, which was used in place of EGFP to allow better spectral separation. Additionally, to ensure a proper detection of viral PICs within the nucleus, cells were immunostained with the nuclear lamin A/C. Cells were then analyzed by confocal microscopy as described above, by recording the three spectral channels corresponding to IN-ECFP, H2B-EYFP and lamin A/C staining (Alexa-680). Figure 7A shows four representative HeLa-H2B-EYFP (red) nuclei delimited by the lamin A/C immunostaining (blue) and containing individual HIV-IN-ECFP viral PICs (green). The observed heterogeneity of the H2B-EYFP fluorescence signal (red) was correlated with PICs localization. A total of 103 PICs were identified in the nuclei of 70 randomly chosen cells and a region of interest (ROI) was defined around each PIC (PIC ROIs). In addition, the same number of ROIs was randomly chosen within the same nuclear planes (Random ROIs). Subsequently, H2B-EYFP fluorescence intensity was measured in the PIC ROIs (Fig. 7B, red bars) and compared to the H2B-EYFP fluorescence intensity in the Random ROIs (Fig. 7B, grey bars). To obtain comparable values among different cells, the H2B-EYFP fluorescence intensity was rescaled over the entire 8-bit range (0–255 a.u.) as described in Methods. As a result of this analysis the average H2B-EYFP fluorescence intensity was equal to 104.5±11 (mean±SD) and 132.6±15 (mean±SD) for PIC ROIs and Random ROIs, respectively. The two distributions were then compared using the non-parametric two-tailed Kolmogorov-Smirnov test yielding a statistically significant difference (*P*<0.001). These results demonstrate that PICs are non-randomly distributed in the nuclei, showing a preferential localization in low H2B-EYFP fluorescence intensity areas, hence in less condensed chromatin structures.

In order to better characterize the chromatin regions occupied by the HIV-IN-ECFP virions, the H2B-EYFP fluorescence intensity was cross analyzed by immunofluorescence with antibodies specific for the tri-methylated K9 of histone H3, a post-translational modification identifying the transcriptionally silenced heterochromatin [31]. This analysis revealed that the average H2B-EYFP fluorescence intensity of the overlapping regions is equal to 195 a.u. and its distribution width at half-height ranges from 140 to 230 as indicated by the arrow in Fig. 7B. Therefore, these data demonstrate that the high-intensity H2B-EYFP fluorescence regions, avoided by viral PICs, are indeed occupied by silenced heterochromatin. Interestingly, a preferential distribution of intranuclear viral PICs was observed toward the periphery of the nuclei. In fact, by measuring the PICs distances from the nuclear lamina, we observed that the majority of IN-EGFP virions localize at distances ranging from 0.4 to 2 µm from the nuclear border, while the frequency of HIV-IN-EGFP PICs decreases dramatically toward the center of the nuclei (Fig. 7C).

Taken together, these results indicate that viral PICs (i) are decisively excluded from heterochromatin regions, and (ii) frequently localize in the less condensed chromatin regions placed in proximity to the inner nuclear envelope.

## Discussion

Integration of the viral cDNA into the host cellular genome is a necessary and intensively investigated event in retroviral replication, particularly as concerns the identification of the preferred integration sites. To date, however, no specific genomic sequences have been associated to such sites. Yet, unraveling the mechanisms of integration-site selection for HIV-1 is important not only to better understand the biology of retroviruses but also because of its impact on other fields as retrovirus-based technology. For instance, retroviral vectors have been extensively developed for gene therapy applications, however their use is limited by the uncontrollable integration site, a situation that may eventually cause disruption of normal cellular proliferation [32]. In recent years wide genome and transcriptome surveys revealed that retroviral integration is favored near transcriptionally active genes [11]–[16]. Mounting evidence suggest that the cellular lens epithelium-derived growth factor (LEDGF/p75), a IN-interacting factor [33], may direct viral integration into transcription units [34], [35]. It has been speculated that LEDGF/p75 may tether PICs at specific sites by interacting through its PWWP domain with specific histone modifications associated with transcriptional elongation. Indeed, the role of the chromatin structure and of higher order nuclear organization in retroviral integration has not been yet investigated.

In this study we observed that chromatin structure is clearly a determinant element for PICs distribution. Indeed, by using HeLa-H2B-EYFP cells as an experimental system to distinguish different chromatin structure regions, this study demonstrates that the vast majority of PICs localize in decondensed regions of the chromatin, as compared to more condensed areas. To better correlate chromatin condensation forms observed in these cells with functionally distinct regions of euchromatin or heterochromatin, we exploited antibodies against post-translational modification of histone H3 (K9 tri-methylated) specific for transcriptionally silenced regions of the heterochromatin [31]. Our data clearly show that PICs are excluded from transcriptionally silenced heterochromatin. Localization in euchromatin regions is in accordance with a recent report showing that proviral integration sites are characterized by specific epigenetic codes [36]. The association with definite regions of the chromatin marked by specific post-translational modifications might also suggest that chromatin modification factor/s might tether HIV-1 to specific sites. We have recently demonstrated that cellular histone-acetyltransferase, p300, acetylates IN and enhances viral integration [37]. This cellular enzyme, which determines chromatin decondensation through histones acetylation, may favor integration by both acetylating IN and by tethering the virus to acetylated/decondensed regions of the chromatin. Further study must be carried out to address these hypotheses [32]. Notably, our results along with published data might also suggest that in addition to specific factors, chromatin structure as such might affect PICs distribution, hence viral integration. One could speculate that PICs are excluded from the heterochromatin regions for physical reasons, since this chromatin form is characterized by pore sizes smaller than 16–20 nm [38] , as opposed to the 30 nm size of the retroviral PICs [1]. DNA-related metabolic processes including transcription, recombination, DNA repair and replication, are coordinated by functionally distinct chromatin domains spatially arranged within a precisely defined nuclear architecture [39]. The analysis of HIV-IN-EGFP PICs immediately revealed a clear preferential position at the nuclear periphery. Interestingly recent advances highlight the role of nuclear envelope components in the control of gene expression (for review, see [40]) thus suggesting that the same nuclear structure may affect the final site of viral integration.

So far, studies aimed at elucidating integration specificity were largely based on genomic screening and molecular biology methods. In this study we have developed and exploited a fluorescently labeled HIV-1 virus that allowed us to study viral PICs distribution within intact nuclei by means of advanced fluorescence microscopy imaging. Important results were previously obtained in understanding virus-host cell interactions by tagging viral-particles with fluorophores linked to viral proteins undetectable at the nuclear levels [21] Only very recently, Arhel et al have reported the development of a system allowing HIV-1 nuclear visualization [41]. However, the tetracystein-labeling approach detects only rare PICs within the nucleus and this low sensitivity, coupled to a fixed analysis time (24 hours in that case, whereas the time resolved analysis reported in Fig. 6C shows a clear prevalence of PICs at 6 hours) hampers the possibility to observe any preferential localization. In the present study the nuclear localization of PICs was carefully analyzed in infected cells immunostained with lamin A/C antibodies in order to precisely define the nuclear compartment. Nuclei were analyzed in a confocal system at high resolution and imaged in the x-y and x-z planes. The combination of multidirectional imaging and accurate image processing allowed us to unambiguously identify the viral particles located inside the nuclear envelope and distinguish them from those located in its outer proximity. In fact, infected cells showed a high number or fluorescent PICs attached to the nuclear membrane, thus an accurate analysis is strictly necessary to establish the nuclear nature of viral PICs. Incidentally, the high frequency of PICs adherent to the borders of the nuclei suggests that the transition through the nuclear membrane is a strong limiting factor during viral replication; an observation also supported by the disproportionate number of nuclear versus cytoplasmic viral particles (1/50 ratio), as similarly reported by others [25].

In conclusion, our analysis demonstrated a structured distribution with respect to the nuclear architecture. We believe that the relatively early preferential localization in euchromatin regions at the periphery of the nuclei shown here provides a first indication of a possible role of the subnuclear structures peculiarities in addressing the viral genomes towards preferential genome domains and might offer useful hints for further in depth investigation of the mechanisms regulating retroviral integration.

## Methods

### Cells and antibodies

HeLa, 293T and HeLa-H2B-EYFP cells (generously supplied by Jörg Langowski) were maintained in DMEM supplemented with 10% FCS. HeLa-H2B-EYFP cells were cultured in medium containing 500 µg/ml of G418 (Gibco BRL, Milan, Italy). Primary antibodies used for immunofluorescence were: mouse mAb AG3.0 anti-HIV p24^CA^ and rabbit anti-HIV p17^MA^ (AIDS Research and Reference Reagent Program), goat anti-Lamin A/C (Santa Cruz Biotechnology, Inc., Santa Cruz, CA), rabbit anti-trimethyl-Histone H3 Lysine 9 (Upstate Biotechnology, NY, USA). Primary antibodies used for western blot analysis were: mouse anti-IN 8G4 obtained from the AIDS Research and Reference Reagent Program and anti-HIV-1 human sera generously supplied by Maurizio Federico. Secondary antibodies used for immunofluorescence were: anti-rabbit or anti mouse conjugated with Alexa-594, Alexa-633 and Alexa-647 (Molecular Probes, Eugene, OR) and anti-goat conjugated with Alexa-680 (Molecular Probes, Eugene, OR). Secondary antibodies used for western blot analysis were: anti-mouse and anti-human conjugated with HRP (Santa Cruz Biotechnology, Inc., Santa Cruz, CA).

### Expression plasmids

pVpr-IN-ECFP was constructed by cloning Vpr (PCR amplified from pNL4.3) in frame with the codon optimized IN [42] into the pECFP-N1 vector (Clontech Laboratories, Inc., Saint-Germain-en-Laye, France) In addition an HIV-1 protease cleavage site (IRKVL), flanked at both C- and N- terminus by a flexible linker (KRIQST), was introduced between Vpr and IN. pVpr-IN-EGFP was constructed by substituting the ECFP cDNA with EGFP. The pD64E and pNL4-3.Luc.R-E- were obtained from the AIDS Reference and Reagent Program.

### Virus Production and Infection

HIV-IN-EGFP (-ECFP) virions were produced by transfecting 3×10^6^ 293T cells by calcium phosphate with 6 µg of pVpr-IN-EGFP (-ECFP), 6 µg of pD64E and 1 µg of pVSV-G. The control viruses were produced by transfecting 3×10^6^ 293T cells by calcium phosphate with 6 µg pNL4-3.Luc.R-E- or pD64E together with 1 µg pVSV-G. Supernatants were collected after 48 hrs, filtered through a 0.45 µm pore size filter and then concentrated by ultracentrifugation. For visualization experiments, luciferase assay and Alu-PCR ultracentrifugation was performed at 110.000× *g* for 2 hrs at 4°C; for western blot analysis at 35,000× *g* for 1.5 hrs at 4°C on a 20% sucrose cushion. Viral titers were quantified by RT assay or p24 ELISA (Innogenetics, Gent, Belgium). Infections for Alu-PCR and luciferase assays were performed using viral loads equivalent to 157.000 RT cpm on 400.000 cells. For immunofluorescence analysis viral loads equivalent to 1,5 µg or 3 µg of HIV-1-p24 were used to infect 40.000 cells; 2 hrs following infection cells were washed and incubated for 1 min with 1× Trypsin (Sigma, Milan, Itlay) to eliminate un-entered virions absorbed onto cellular membrane.

Alu-PCR was performed as described in [37]

### Immunofluorescence and NERT fluorescence labeling

Viral particles immunostaining was performed by adsorbing viral supernatants on chamber slides (BD Biosciences, Bedford, MA, USA) for 4 hrs at 37°C with 10 µg/ml of polybrene, followed by rinsing with phosphate-buffered saline (PBS) and fixation with 2% paraformaldehyde in PBS for 15 minutes at room temperature. Intracellular immunostaining was performed in cells grown on chamber slides. Coverslips were then fixed with 2% paraformaldehyde in PBS for 15 minutes at room temperature followed by incubation for 5 minutes with glycine 100 mM in PBS and permeabilization with 0,1% Triton X-100 in PBS for 5 minutes. After treatment in blocking buffer (0,1% Tween and 1% BSA in PBS) for 30 minutes, primary antibodies were incubated for 1 hour at 37°C in blocking buffer followed by incubation for 1 hr at 37°C with secondary antibodies. Chamber slides were mounted with Vectashield before microscopy analysis (Vector Laboratories Inc., Burlingame, CA).

Virions containing fluorescently labeled cDNA by NERT activity were prepared by incubating 400 ng of p24 viral supernatants for 4 hrs at 37°C with an endogenous reverse transcription reaction buffer, as previously described [23], [24] and modified as follows: 10 mM Tris-HCl (pH7.4), 150 mM NaCl, 1 mM MgCl_2_, 100 µM dATP, 100 µM dCTP, 100 µM dGTP, 25 µM dUTP, 10 µM Alexa-594-dUTP (Molecular Probes, Eugene, OR).

### Image acquisition and analysis

Three-dimensional stacks of fixed cells were acquired with the TCS SL laser-scanning confocal microscope (Leica Microsystems, Milan, Italy) equipped with galvanometric stage using a 63×/1.4 NA HCX PL APO oil immersion objective. Z-step and y-step size was 0.3 µm. An Ar laser was used for ECFP (λ = 458 nm), EGFP (λ = 488 nm), EYFP (λ = 514 nm) and Alexa-680 (λ = 633 nm) excitation. Fluorescence emission was collected in the ranges 468–494, 495–525, 527–585 and 587–722 nm for ECFP, EGFP, EYFP and Alexa-680, respectively. For the two- and three-color analysis a sequential image acquisition was used to reduce crosstalk between different signals below 5%. Multi-channel images were contrast stretched (linearly) and assembled in ImageJ (NIH). H2B-EYFP fluorescence intensity among different cell images were normalized using the following procedure: for the acquisition the laser power was adjusted to maximize dynamic range and to avoid image saturation with the brightest value ranging between 220 and 250; the contrast range was then linearly expanded assigning the brightest value to 255. For cross analysis with 3MeK9H3 staining the H2B-EYFP fluorescence intensity was quantified in the 3MeK9H3 positive regions after rescaling with the above-described procedure.

Raw data (i.e. confocal z stacks) were deconvolved using the experimental PSF measured for each channel and imposing the optical parameters adopted during image acquisition. For each fluorescent channel the point spread function (PSF) of the microscope was calculated using PSF distilled function in Huygens Essential software (Scientific Volume Imaging BV. Hilversum, The Netherlands). There the lamin A/C Alexa-680 signal (blue) was linearly expanded between 30 and 250, while the PICs signal (green) between 2 and 50. The applied lookup table defines a grey background for values below the cutoff = 30.

## Supporting Information

Figure S1 Vertical y stack series of nuclear HIV-IN-EGFP PICs. Vertical y stack of a HeLa cell nucleus infected with IN-EGFP virions (green) and immunostained with lamin A/C antibody (blue). A distance of 0.3 µm separates adjacent frames. White arrows indicate a single viral PIC identified as intranuclear and visible in two subsequent sections. Bars, 5 µm.
(11.60 MB DOC)
