# Supplementary material for: HIV-1 Pre-Integration Complexes Selectively Target Decondensed Chromatin in the Nuclear Periphery
Source: PLoS One. 2008 Jun 11;3(6):e2413. doi: 10.1371/journal.pone.0002413 (PMC2398779; doi:10.1371/journal.pone.0002413)
Supplement: Figure S1 — Vertical y stack series of nuclear HIV-IN-EGFP PICs. Vertical y stack of a HeLa cell nucleus infected with IN-EGFP virions (green) and immunostained with lamin A/C antibody (blue). A distance of 0.3 µm separates adjacent frames. White arrows indicate a single viral PIC identified as intranuclear and visible in two subsequent sections. Bars, 5 µm. (11.60 MB DOC) [file pone.0002413.s001.doc]

**SUPPORTING INFORMATION**

**Vertical y stack series of HIV-IN-EGFP PICs**

It is worth noting that during our analysis several PICs were detected outside the nucleus and in particular adjacent to the nuclear surfaces leading to misinterpret the fluorescent signals as originating from within the nuclear membrane. Conversely, the actual intranuclear PICs localization can be effectively probed by imaging vertical sections of the cell under study allowing identifying directly PICs located inside the nucleus and distinguishing them from those located in its outer proximity. Such a vertical stack is shown in Fig. S1, which reveals the presence of a number of PICs outside the nucleus, very often attached to the nuclear membrane. The presence of a single PIC within the nuclear compartment is indicated in two subsequent sections by white arrows.

**Figure S1. Vertical y stack**

Vertical y stack of a HeLa cell nucleus infected with IN-EGFP virions (green) and immunostained with lamin A/C antibody (blue). A distance of 0.3 μm separates adjacent frames. White arrows indicate a single viral PIC identified as intranuclear and visible in two subsequent sections. Bars, 5 μm.

**
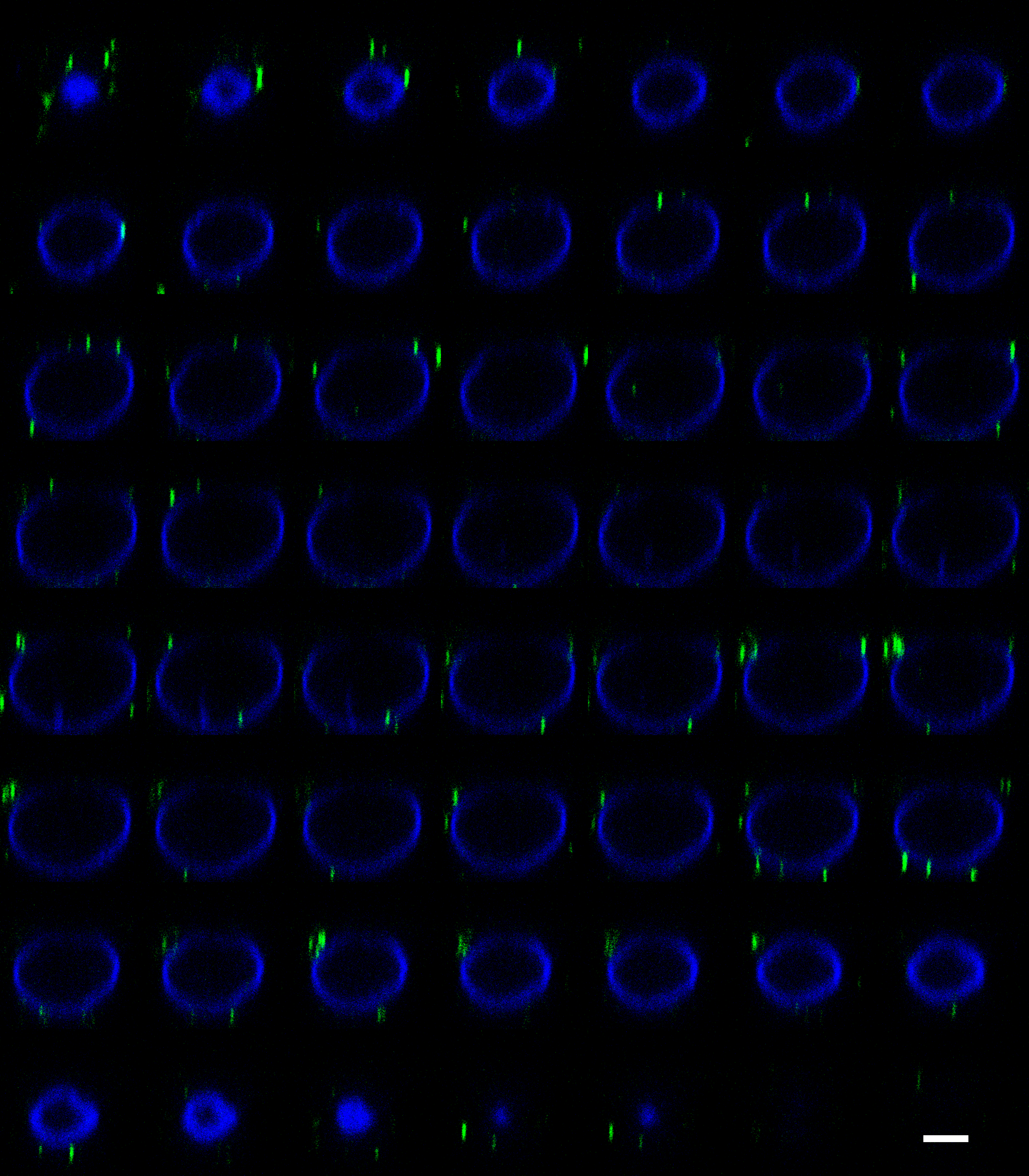
**
